# Adding salt to food at table as an indicator of gastric cancer risk among adults: a prospective study

**DOI:** 10.1007/s10120-024-01502-9

**Published:** 2024-04-17

**Authors:** Selma Kronsteiner-Gicevic, Alysha S. Thompson, Martina Gaggl, William Bell, Aedín Cassidy, Tilman Kühn

**Affiliations:** 1 Department of Nutritional Sciences, University of Vienna, Josef-Holaubek-Platz 2 (UZA II), 1090 Vienna, Austria; 2 Center for Public Health, Medical University of Vienna, Kinderspitalgasse 15, 1090 Vienna, Austria; 3 Institute for Global Food Security, School of Biological Sciences, Queen’s University Belfast, 19 Chlorine Gardens, Belfast, BT9 5DL UK

**Keywords:** Sodium chloride, Stomach neoplasms, Diet

## Abstract

**Background:**

While dietary salt intake has been linked with gastric cancer risk in Asian studies, findings from Western populations are sparse and limited to case—control studies. Our aim was to evaluate the frequency of adding salt to food at table in relation to gastric cancer risk among UK adults.

**Methods:**

We evaluated associations between the frequency of adding salt to food and the risk of gastric cancer in the UK Biobank (N = 471,144) using multivariable Cox regression. Frequency of adding salt to food was obtained from a touchscreen questionnaire completed at baseline (2006–2010). 24-h urinary sodium excretion was estimated using INTERSALT formulae. Cancer incidence was obtained by linkage to national cancer registries.

**Results:**

During a median follow-up period of 10.9 years, 640 gastric cancer cases were recorded. In multivariable models, the gastric cancer risk among participants reporting adding salt to food at table “always” compared to those who responded “never/rarely” was HR = 1.41 (95% CI: 1.04, 1.90). There was a positive linear association between estimated 24-h urinary sodium levels and the frequency of adding salt to food (p-trend <0 .001). However, no significant association between estimated 24-h urinary sodium with gastric cancer was observed (HR = 1.19 (95% CI: 0.87, 1.61)).

**Conclusions:**

“Always adding salt to food” at table was associated with a higher gastric cancer risk in a large sample of UK adults. High frequency of adding salt to food at table can potentially serve as a useful indicator of salt intake for surveillance purposes and a basis for devising easy-to-understand public health messages.

**Supplementary Information:**

The online version contains supplementary material available at 10.1007/s10120-024-01502-9.

## Introduction

Gastric cancer is the fifth most commonly diagnosed cancer globally [1], with the highest prevalence in Asia, followed by Eastern Europe and Latin Americas [1–3]. Gastric cancer risk increases with age and is more prevalent in males, while other factors that increase risk include tobacco and alcohol use, *Helicobacter pylori* (*H. pylori*) infection, consumption of some Asian-style salt-preserved foods, and being overweight/obese [4–7]. After initial declines in gastric cancer rates in the second half of the past century, recent studies are raising concerns as incidence rates are now increasing among young adults (< 50 years) globally [2, 8–10].

The role of dietary salt intake in gastric cancer risk has been widely investigated, yet the evidence is inconclusive [11, 12]. Some studies hypothesize its role in disrupting stomach mucosa and making it more susceptible to *H. pylori* colonization [4, 13]. Salt may also increase gastric cancer risk via mechanisms independent of *H. pylori* infection, for example by damaging the gastric epithelium in synergy with N-nitroso compounds and chemical carcinogens [14]. While there is strong evidence on the role of consumption of some salt-preserved foods in gastric cancer aetiology, data on intakes of added or total salt yielded mixed results, with the majority of studies conducted in Asian populations with higher gastric cancer prevalence [12]. This may partly be attributed to methodological issues in measuring dietary salt intake [15]; repeated 24-h urinary sodium excretion, the “gold standard” method for estimating sodium intake is expensive and burdensome, making it impractical for use in large study samples required for evaluating associations with rare outcomes; using spot urine samples to estimate salt exposure, on the other hand, is prone to random error due to variation in sodium intake and hence urinary sodium excretion. While a number of formulae for estimating 24-h urinary sodium from spot urines, including INTERSALT, Kawasaki and Tanaka, are widely used as indicators of sodium intake, they have been criticized for differential misclassification (e.g. overestimating intake in some and underestimating in other population groups) [15–17]. Finally, it is difficult to accurately quantify sodium intake with self-report diet assessment instruments such as 24-h diet recalls or food frequency questionnaires (FFQs) due to a highly variable sodium content in packaged products and respondent difficulty in estimating meal portion sizes and the amount of salt added during cooking [18, 19].

Self-reported data on “adding salt to food” at table may potentially serve as a simple measure of habitual salt intake not as influenced by day-to-day variation in intakes, and one that may be converted to an easy-to-understand-and-apply public health message. In a recent study of UK Biobank participants, high frequency of adding salt to food at table was associated with higher spot urine sodium concentrations and with a higher risk of premature mortality [20]. As only a few prospective studies have evaluated associations between salt intake and gastric cancer [11], with the majority of findings coming from Asian populations, we investigated the association between adding salt to food at table and the risk of stomach cancer in a large prospective study of UK adults. In ancillary analyses, we evaluated associations of spot urinary sodium with the risk of gastric cancer, and associations between the frequency of adding salt to food at table and other indicators of sodium intake—urinary and total dietary sodium. The latter analyses were carried out to evaluate whether adding salt to food at table can be used as a more a simple indicator of sodium exposure in research and clinical practice.

## Methods

### Study population

We used data from the UK Biobank, a large population-based prospective study of UK adults that has been described in detail elsewhere [21]. In our study, data of 502,367 participants were available for analysis. 27,085 study participants with prevalent cancer at baseline (any cancer except for non-melanoma skin cancer), and 3308 participants reporting having existing kidney disease (self-reported during verbal interview) were excluded (Supplemental Fig. 1). We also excluded 1082 participants for whom data on adding salt to food was missing (either missing at baseline or ‘prefer not to answer’) and 20,469 participants with missing data on urinary sodium or potassium levels, or missing baseline BMI (required for calculating INTERSALT formulae, see below). Thus, our analyses of associations between adding salt to food at table and gastric cancer included 471,144 participants, while those with urinary sodium were restricted to 451,757 participants. The UK Biobank study was approved by the National Health Service (NHS) North West Multicentre Research Ethics Committee. All participants provided written informed consent at recruitment. The present study was conducted under the UK Biobank application number 64426.

### Exposure, covariate and outcome assessment

The exposure was ascertained using a variable on adding salt to food at table measured by the baseline touch-screen questionnaire (2006–2010, Data-Field 1478). Participants were asked: “Do you add salt to your food? (Do not include salt used in cooking)” and could answer: “never/rarely”, “sometimes”, “usually”, “always” and “prefer not to answer”. Urinary sodium, creatinine and potassium were measured at baseline from random spot urine samples by the Ion Selective Electrode method using Beckman Coulter AU5400, UK Ltd [22]. We estimated 24-h urinary sodium using INTERSALT equations for men and women [23, 24]. The INTERSALT formulae were chosen as they include population-specific intercepts (including those for Western populations) [24], take into consideration sex-specific differences in sodium excretion, and include potassium as important factor in sodium excretion [25].

Sociodemographic (sex, age, ethnicity, education level, Townsend deprivation index) and lifestyle (diet, smoking, alcohol use, physical activity) covariate data were obtained from the baseline touch-screen questionnaire. Body mass index (BMI) was calculated from weight and height measured by trained staff at recruitment; use of diuretics was reported by participants during verbal interview at baseline. Multimorbidity was estimated using a previously described approach [26] [27]. *H. pylori* infection status was obtained from verbal interview at baseline (self-reported) and hospital impatient data.

Finally, gastric cancer status was ascertained by linkage to the national cancer registries (ICD-10, C16.0–C16.9). Cancer diagnosis data were provided through record linkage to National Cancer Registries in England, Wales (follow-up data available from the NHS Information Centre until February 2020), and Scotland (follow-up data available from the NHS Central Register of Scotland until January 2021). All cardia and non-cardia cases were included. Due to a small number of cases by site, all gastric cancer cases were pulled together for the main analysis.

### Statistical analysis

Associations between the frequency of adding salt to food at table and gastric cancer risk were assessed using Cox proportional hazards models with age as the time scale. Age at baseline was defined as age at study entry, and age at gastric cancer diagnosis, death, or latest follow-up (whichever occurred first) was used as age at exit. Models were first adjusted for sex and ethnicity (White, Mixed, Asian or Asian British, Black or Black British, other, missing (0.4%)), with multivariable-adjusted models further including education level (low, medium, high, missing (19%)), Townsend deprivation index (in quintiles, missing (0.1%)), smoking status (never, previous, current, missing (19%)), body mass index (< 18.5, ≥ 18.5– < 25, ≥ 25– < 30, ≥ 30, missing (0.5%)), physical activity level (MET hours/week, in tertiles, missing (4%)), alcohol consumption (< 1 g/d, 1–7 g/d, 8–15 g/d, ≥ 16 g/d, missing (24%)), use of diuretics (yes/no), and multimorbidity (number of prevalent long-term conditions: 0, 1, 2, or 3 and more). Finally, models were adjusted for dietary factors obtained at baseline (beef intake, pork intake, processed meat intake, fresh fruit intake, salad/raw vegetable intake, and cooked vegetable intake). For use in models, we summed up beef, pork and processed meat intake as “red meat intake” and fruit and vegetable intakes into “fruit and vegetable intake” and categorized these scores into tertiles. Cox regression models with urinary sodium as the exposure (tertiles) based on the INTERSALT formulae were fitted using the similar multivariable models as for added salt, albeit without adjustment for BMI, as it was already included in the formulae. Linear trend tests were carried out by modeling the added salt and the estimated 24-h urinary sodium as continuous variables. We also evaluated the associations between frequency of adding salt to food at table with spot urinary sodium and estimated 24-h urinary sodium concentrations. To test whether adding salt to food is indicative of total dietary salt intake we used data from a subset of 198,900 UK Biobank participants who provided at least one 24-h dietary recall 2.4 (± 1.4) years after baseline.

In sensitivity analyses, we excluded the first year of follow-up, participants with multimorbidity (two or more long-term conditions), participants with ethnic background other than White, and participants with a positive *H. pylori* infection status. To evaluate heterogeneity by cancer site, we fitted separate models for cardia (N = 264) and non-cardia (N = 163) cases the frequency of adding salt to food as a continuous exposure, followed by Cochran’s Q test for heterogeneity.

Statistical analyses were conducted using SAS version 9.4 (SAS Institute Inc). All statistical tests were two sided, and P < 0.05 was considered to be statistically significant.

## Results

### Characteristics of the study population

Over 10.9 years of follow-up, 640 incident gastric cancer cases were recorded from our 471,144 participants. Participants (Table 1) reporting “always” adding salt to food at table were more likely to be male, non-white, to have a lower education level and a higher Townsend deprivation index; they were more likely to be a past/current smoker, and to have high alcohol (≥ 16 g of ethanol/day) intakes.

**Table 1:** Study sample characteristics at baseline in 2006–2010 by the frequency of adding salt to food at table (N = 471,144)

	Total	Frequency of adding salt to food at table			
		Rarely/never (N = 261,195)	Sometimes (N = 132,238)	Usually (N = 54,753)	Always (N = 22,958)
Age, years (mean, sd)	56.3 (8.1)	56.3 (8.1)	56.2 (8.1)	56.8 (8.1)	55.7 (8.3)
Sex, male (%)	46.1	44.5	46.5	51.7	48.9
Ethnicity, white (%)	90.5	91.8	89.9	88.3	83.8
Education level, medium/high (%)	56.3	57.6	57.5	54.6	45.0
Townsend deprivation index (mean, sd)	−1.3 (3.1)	−1.5 (3.0)	−1.2 (3.0)	−1.1 (3.2)	−0.2 (3.5)
Alcohol consumption, 16 + grams of ethanol/day (%)	32.3	29.3	33.9	39.9	39.9
Smoking status, previous/current (%)	44.9	40.4	47.2	54.4	59.1
BMI, kg/m^2^ (mean, sd)	27.4 (4.8)	27.2 (4.7)	27.6 (4.8)	27.8 (4.8)	28.1 (5.1)
Physical activity, METs hr/week (mean, sd)	35.0 (49.1)	34.7 (47.2)	34.9 (49.3)	35.1 (50.6)	38.9 (62.7)
Use of diuretics, yes (%)	7.6	8.0	7.2	7.1	7.1
*H. pylori* infection, yes (%)^a^	0.3	0.3	0.3	0.4	0.4
Multimorbidity, 2 or more (%)	29.7	29.3	29.4	30.9	32.4
Spot urine sodium excretion (mmol/L) (mean, sd)	77.8 (44.6)	73.3 (42.7)	80.8 (45.0)	86.0 (46.9)	92.7 (84.7)
Estimated 24-h sodium excretion using INTERSALT formulae (g/d) (mean, sd)	3.0 (2.9)	2.9 (7.7)	3.0 (8.1)	3.1 (8.4)	3.2 (8.5)

^a^*H. pylori* status was ascertained from self-reported and hospital impatient data

### Frequency of adding salt to food and gastric cancer risk

Table 2 shows the main findings of our study. In sex and ethnicity-adjusted models, the hazard ratio (HR) comparing participants who reported “always” adding salt to food at table and participants who reported adding salt to food “never/rarely” (reference group) was HR = 1.88 (95% CI: 1.41, 2.52); in multivariable models this hazard was HR = 1.39 (95% CI: 1.03, 1.87). The results did not considerably change after adjusting for dietary factors; hazard ratio was HR = 1.41 (95% CI: 1.04, 1.91).

**Table 2:** Hazard ratios and 95% confidence intervals for the frequency of adding salt to food at table and the risk of gastric cancer (N = 471,144)

	Never/rarely	Sometimes	Usually	Always	P-trend
Cases, n	322	175	90	53	
Person-years	2,746,652.9	1,390,018.7	573,214.5	238,256.2	
Sex and ethnicity-adjusted	1 (ref)	1.07 (0.89, 1.28)	1.21 (0.96, 1.53)	1.88 (1.41, 2.52)	0.0002
Multivariable-adjusted^a^	1 (ref)	0.99 (0.82, 1.19)	1.06 (0.84, 1.34)	1.39 (1.03, 1.87)	0.10
Multivariable-adjusted + dietary factors^b^	1 (ref)	0.99 (0.82, 1.19)	1.06 (0.84, 1.35)	1.41 (1.04, 1.90)	0.09
Excluding the first year of follow-up^c^	1 (ref)	0.95 (0.79, 1.15)	1.04 (0.82, 1.33)	1.46 (1.08, 1.97)	0.09
Excluding participants with multimorbidity^d^	1 (ref)	0.98 (0.77, 1.23)	1.13 (0.84, 1.51)	1.33 (0.91, 1.95)	0.17
Excluding participants with *H. pylori* infection^e^	1 (ref)	1.00 (0.93, 1.20)	1.07 (0.85, 1.36)	1.39 (1.03, 1.88)	0.09
Restricting the sample to White ethnic background^f^	1 (ref)	0.97 (0.80, 1.18)	1.06 (0.83, 1.36)	1.35 (0.98, 1.86)	0.24

^a^Sex, ethnicity (White, Mixed, Asian or Asian British, Black or Black British, other, missing), education level (low, medium, high), Townsend deprivation index (in quintiles, missing), smoking status (never, previous, current, missing), alcohol consumption (< 1 g/day, 1-7 g/d, 8-15 g/d, 16 + g/d, missing), physical activity (in MET hours/week, categorized into tertiles, missing), BMI (< 18.5, ≥ 18.5- < 25, ≥ 25- < 30, ≥ 30, missing), use of diuretics (yes, no), multimorbidity (0, 1, 2, 3 +)

^b^Also adjusted for dietary factors at baseline (frequency of consumption of beef, processed meat, pork, fresh fruit, salad/raw vegetables, and cooked vegetables, in tertiles)

^c^Excluding the first year of follow-up in multivariable + dietary factors’ models. N = 470,383; N_cases_ = 602

^d^Excluding participants with multimorbidity at baseline (i.e. persons with 2 or more coexisting disease conditions) in multivariable + dietary factors’ models. N = 331,006; N_cases_ = 408

^e^Excluding participants with *H. pylori* infection in multivariable + dietary factors’ models. *H. pylori* status was ascertained from self-reported and hospital impatient data. N = 469,180; N_cases_ = 637

^f^Restricting the sample to participants who reported their ethnicity as “White”. N = 426,317; N_cases_ = 585

In sensitivity analyses, hazard ratios in models adjusted for dietary factors were: HR = 1.46 (95% CI: 1.08, 1.97) after excluding the first year of follow-up; HR = 1.33 (95% CI: 0.91, 1.95) after excluding participants with multimorbidity; and HR = 1.39 (95% CI: 1.03, 1.88) after excluding persons with *H. pylori* infection at baseline. When we restricted the analysis to only White participants, the hazard ratio was HR = 1.40 (95% CI: 1.01, 1.93). No evidence of heterogeneity by cancer site was found, with hazard ratios for cardia cases and non-cardia cases being HR = 1.01 (95% CI: 0.88, 1.15) and HR = 1.09 (95% CI: 0.93, 1.27) (P = 0.75), respectively.

### Urinary sodium and gastric cancer risk

Although the HR comparing the top tertile (T3) with the lowest tertile (T1) of estimated 24-h urinary sodium and gastric cancer was HR = 1.40 (95% CI: 1.03, 1.90) in age and ethnicity-adjusted models was (Table 3) this was no longer statistically significant following multivariable-adjustment (HR = 1.19 (95% CI: 0.87, 1.61).

**Table 3:** Hazard ratios and 95% confidence intervals of INTERSALT formula-estimated 24-h urinary sodium and gastric cancer risk (N = 451,757)

Estimation method	T1^a^	T2	T3	P-trend
Cases, n	139	144	329	
Sex and ethnicity-adjusted	1 (ref)	0.98 (0.74, 1.29)	1.40 (1.03, 1.90)	0.007
Multivariable-adjusted^b^	1 (ref)	0.91 (0.70, 1.19)	1.19 (0.87, 1.61)	0.11
Excluding the first year of follow-up^c^	1 (ref)	0.93 (0.70, 1.24)	1.29 (0.94, 1.77)	0.04
Excluding participants with multimorbidity^d^	1 (ref)	0.89 (0.63, 1.25)	1.13 (0.77, 1.66)	0.28
Excluding participants with *H. pylori* infection^e^	1 (ref)	0.91 (0.70, 1.20)	1.19 (0.88, 1.61)	0.12
Restricting the sample to White ethnic background^f^	1 (ref)	0.92 (0.69, 1.23)	1.18 (0.86, 1.63)	0.15

^a^Tertile of estimated 24-h urinary sodium excretion

^b^Sex, ethnicity (White, Mixed, Asian or Asian British, Black or Black British, other, missing), education level (low, medium, high), Townsend deprivation index (in quintiles, missing), smoking status (never, previous, current, missing), alcohol consumption (< 1 g/day, 1-7 g/d, 8-15 g/d, 16 + g/d, missing), physical activity (in MET hours/week, categorized into tertiles, missing), use of diuretics (yes/no), and multimorbidity (0, 1, 2, 3 +)

^c^Excluding the first year of follow-up in multivariable models. N = 451,068; N_cases_ = 576

^d^Excluding participants with multimorbidity at baseline (i.e. persons with 2 or more coexisting disease conditions) in multivariable models. N = 318,117; N_cases_ = 393

^e^Excluding participants with *H. pylori* infection in multivariable models. *H. pylori* status was ascertained from self-reported and hospital impatient data. N = 450,485; N_cases_ = 609

^f^Restricting the sample to participants who reported their ethnicity as White. N = 408,711; N_cases_ = 557

After excluding gastric cancer cases occurring in the first year of follow-up the hazard ratio was HR = 1.29 (95% CI: 0.94, 1.77). The results did not materially change with exclusion of participants with baseline *H. pylori* infection with HR = 1.19 (95% CI: 0.88, 1.61), and were somewhat weaker when participants with multimorbidity were excluded, with HR = 1.13 (95% CI: 0.77, 1.66). When participants with non-White background were excluded from the analysis, the hazard ratio was HR = 1.18 (95% CI: 0.86, 1.63).

### Associations of the frequency of adding salt to food with urinary and dietary sodium

**Fig. 1:**
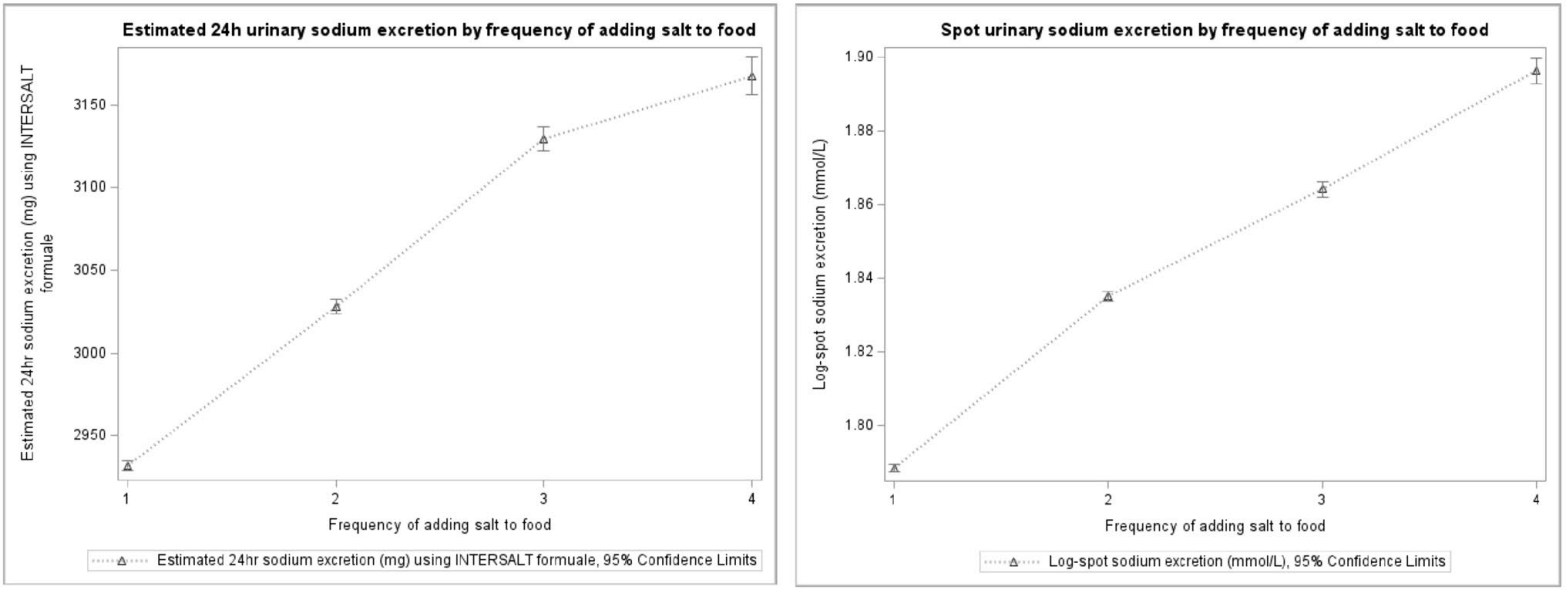
Association between 24-h estimated urinary sodium, spot urinary sodium, and the frequency of adding salt to food at table. Frequency of adding salt to food: 1-never/rarely; 2-sometimes; 3-usually; 4-always. All associations were statistically significant, with p-values for linear trend across categories of adding salt  P< 0.001

Figure 1 shows positive dose—response associations of the self-reported frequencies of adding salt to food at table with the estimated 24-h urinary and spot urinary sodium concentrations. “Never/rarely”, “sometimes”, “usually” and “always” groups corresponded with 2932 (95% CI: 2929, 2935), 3028 (95% CI: 3023, 3032), 3129 (95% CI: 3122, 3136) and 3168 (95% CI: 3157, 3178) mg of urinary sodium per day (P-trend < 0.001). Similarly, concentrations of log-spot urinary sodium across the four frequencies of adding salt to food at table were 1.79 (95% CI: 1.79, 1.79), 1.84 (95% CI: 1.84, 1.84), 1.86 (95% CI: 1.86, 1.87) and 1.90 (95% CI: 1.90, 1.90) mmol/L (P-trend < 0.001).

In a subset of 198,900 participants with data on sodium intake from 24-h recall carried out 2.4 (± 1.4) years after the baseline (Supplemental Fig. 2), a positive association between the frequencies of adding salt to food at table and the estimated dietary sodium intake was observed. “Never/rarely”, “sometimes”, “usually” and “always” groups corresponded with 1864 (95% CI: 1860, 1869), 2040 (95% CI: 2034, 2047), 2,196 (95% CI: 2186, 2206) and 2254, (95% CI: 2235, 2272) mg of dietary sodium per day (P-trend < 0.001).

## Discussion

In this prospective study of UK Biobank participants, we found that participants who always added salt to food at table had a 41% greater risk of developing gastric cancer than those who never/rarely added salt, in models adjusted for demographic, socioeconomic and lifestyle factors, and for prevalent comorbidities. We also found that adding salt to food at table had a positive dose–response association with spot urinary sodium and 24-h urinary sodium excretion estimated by INTERSALT formulae. However, when we used the 24-h urinary sodium as exposure, we did not observe any associations with the gastric cancer risk, likely due to dilution effects that have been previously described in studies comparing sodium exposure based on spot vs. 24-h urine samples [28, 29]. After excluding gastric cancer cases occurring in the first year of follow-up, however, these associations became slightly stronger, suggesting presence of reverse causality (e.g., persons with gastric symptoms preceding cancer diagnosis might have reduced their salt intake) [30].

Our findings on “always adding salt to food” at table and gastric cancer are in line with the findings of a recent pooled analyses of 25 case–control studies conducted in America, Asia and several European countries [31], which showed positive associations between added salt and gastric cancer. They are also in line with findings from a recent meta-analysis of prospective studies showing higher gastric cancer risk among Asian populations with high intakes of salt, salted fish, pickled foods and processed meats, used as proxies of total salt intake among study participants [32]. Two previous smaller studies from Europe, on the other hand, did not show associations between total salt intake derived from dietary questionnaires and gastric cancer risk [33, 34]. These inconsistencies in findings across studies of gastric cancer risk with consumption of salt measured using different indicators (estimated total dietary sodium intake, intake of specific foods with high salt content) may be in part explained by the difficulty of measuring total salt intake by dietary questionnaires [29, 35, 36]. In turn, our study suggests that adding salt to food is an eating behavior that may be a good proxy of habitual salt intake and less prone to day-to-day variation than salt estimated from 24-h diet recalls, as also indicated by the dose—response association we observed between added salt intake and urinary sodium levels. In addition, “always adding salt to food” at table may serve as a simple indicator for estimating excessive sodium in large populations. It is also easily convertible to a public health message, and may aid in reducing overall sodium intake both on individual and population levels.

While our study based on a large cohort suggests that always adding salt to food at table is also associated with a higher risk of gastric cancer in Western populations, it has several limitations. Case numbers in our study were not sufficient to evaluate the influence of potential modifiers such as sex, age, ethnicity, *H. pylori* infection, or smoking status. Analyses stratified by anatomical cancer site were restricted due to low case numbers. While we found no heterogeneity in risk associations between added salt intake and cardia vs. non-cardia gastric cancer, larger studies are needed to assess potential differences across cancer subtypes. *H. pylori* status was ascertained from self-reported and hospital impatient data as data from stool or breath samples were not available in UK Biobank; with 0.3% estimated prevalence among UK Biobank participants vs. 35.5% estimated UK prevalence of *H. Pylori* infection it was most likely underestimated [37]. Also, due to the observational nature of our study residual confounding cannot be excluded. Our ancillary analyses on urinary sodium and gastric cancer were restricted to spot urine samples, which have been shown to lead to biased associations with cardiovascular diseases compared to repeated 24-h urine samples [28, 29], including one study in UK Biobank that did not show significant associations between spot urine sodium and cardiovascular disease risks [38]. In addition, the case number available for these analyses was rather low. Next, while we did not have data on salt intake via foods for the full UK Biobank cohort, although the present analyses in a subset of participants with detailed dietary data indicate that individuals, who add more salt are also more likely to consume foods with higher sodium content; thus, and given that dietary salt intake is prone to measurement error, true associations between salt intake and gastric cancer risk could be stronger than those observed in this study. Finally, our findings cannot be generalized to the general UK population due to voluntary participation and age restriction of the UK Biobank cohort.

To conclude, our prospective study in a large sample of 471,144 UK adults suggests that routinely adding salt to food at table is associated with a greater risk of gastric cancer compared to never/rarely adding salt. More studies on salt intake and gastric cancer risk are needed especially among other non-Asian populations and based on repeated 24- h urinary sodium measurements to better quantify the association between sodium exposure and gastric cancer risk.

### Supplementary Information

Below is the link to the electronic supplementary material.

Supplementary file1 (DOCX 78 KB)
