# Supplementary material for: Adding salt to food at table as an indicator of gastric cancer risk among adults: a prospective study
Source: Gastric Cancer. 2024 Apr 17;27(4):714–21. doi: 10.1007/s10120-024-01502-9 (PMC11193689; doi:10.1007/s10120-024-01502-9)
Supplement: Supplementary file 1 — Supplementary file1 (DOCX 78 KB) [file 10120_2024_1502_MOESM1_ESM.docx]

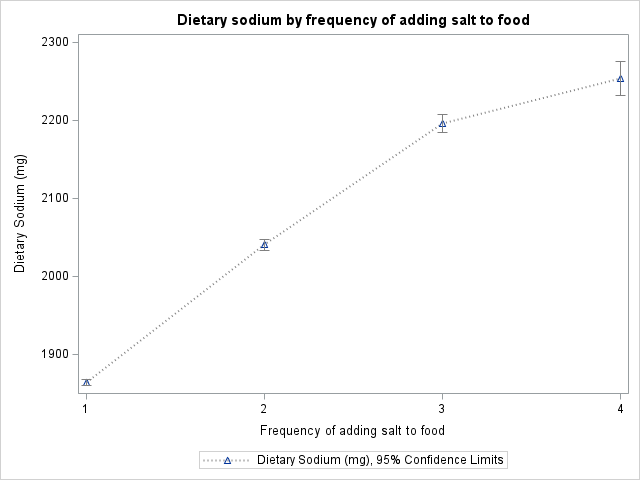


**Supplemental figure 2. Association between dietary sodium estimated from 24-h diet recall and the frequency^a^ of adding salt to food at table**

^a^ Frequency of adding salt to food: 1- never/rarely; 2-sometimes; 3-usually; 4-always. All associations were statistically significant, with P-values for linear trend across categories of adding salt: P<0.001.
